# The Cyclopædia of Practical Surgery

**Published:** 1840-04

**Authors:** 


Art. IX.—*The Cyclopædia of Practical Surgery.* Edited by W. B. Costello, m.d. Parts IV-V.—*London, Jan. March,* 1840.

We are much pleased to find, by the appearance of the present parts, that the publication of this important work, after a long suspension, has recommenced, and is not likely to be again interrupted.

The editor informs us, "that the cause of the delay has been entirely removed, and that in future the parts will be published with punctuality," every alternate month. We are willing to believe the additional assurance of the editor, "that the interval has been profitably occupied in the improvement of the general plan, and in securing the cooperation of additional contributors of the most distinguished reputation." Indeed, if excellence can be assured by numbers or by celebrity, surely the Cyclopædia of Surgery will be excellent, as we think the list of contributors on the cover exceeds, in both respects, that engaged in any previous work of the same kind. To be sure, out of the ninety names we have counted there, some (we shall not say how many) might be dispensed with without detriment to the reputation or probable utility of the work; but there would still remain enow to constitute a phalanx of which surgical science might be justly proud.

The present parts contain 27 articles (*Ankylosis—Burns*), by 17 different writers. They are of various value, but on the whole they are good, and some are excellent. On a former occasion, in noticing this work, we felt it to be our duty to protest against the vitiation of our language, by the unnecessary and improper introduction of foreign words and phrases. We are sorry to say that the Parts before us are not altogether free from the same fault; as where we read, "Every areole or cell of the deep surface of the derm;" "if the inflammatory irritation that assails the cellulo-adipose pellets lodged in the areoles of the derm can be arrested, the disease aborts;" "when the granulations have become *regularized*," &c. We hope the learned editor will see to this, and not forget that he is responsible for the language as well as the subjects of his articles; and that if English surgery does well to search other nations for the materials of its structures, it has at home a language wherewith to build them, that assuredly needs no foreign aid of ornament.

